# High-throughput synthesis of CeO_2_ nanoparticles for transparent nanocomposites repelling *Pseudomonas aeruginosa* biofilms

**DOI:** 10.1038/s41598-022-07833-w

**Published:** 2022-03-10

**Authors:** Massih Sarif, Olga Jegel, Athanasios Gazanis, Jens Hartmann, Sergi Plana-Ruiz, Jan Hilgert, Hajo Frerichs, Melanie Viel, Martin Panthöfer, Ute Kolb, Muhammad Nawaz Tahir, Jörg Schemberg, Michael Kappl, Ralf Heermann, Wolfgang Tremel

**Affiliations:** 1 Department Chemie, Johannes Gutenberg-Universität Mainz, Duesbergweg 10-14, 55128 Mainz, Germany; 2 Institut Für Molekulare Physiologie, Mikrobiologie und Biotechnologie, Johannes-Gutenberg-Universität Mainz, Hanns-Dieter-Hüsch-Weg 17, 55128 Mainz, Germany; 3 Department of Materials and Geoscience, Technical University Darmstadt, Petersenstrasse 23, 64287 Darmstadt, Germany; 4 Chemistry Department, King Fahd University of Petroleum and Materials, Dhahran, 31261 Saudi Arabia; 5 Interdisciplinary Research Center for Hydrogen and Energy Storage (IRC-HES), King Fahd University of Petroleum and & Minerals, Dhahran, 31261 Saudi Arabia; 6 Institut Für Bioprozess-Und Analysenmesstechnik E.V., Rosenhof, 37308 Heilbad Heiligenstadt, Germany; 7 Max-Planck-Institute for Polymer Research, Ackermannweg 10, 55128 Mainz, Germany

**Keywords:** Flow chemistry, Bioinspired materials, Disease prevention, Nanobiotechnology, Nanoscale materials

## Abstract

Preventing bacteria from adhering to material surfaces is an important technical problem and a major cause of infection. One of nature’s defense strategies against bacterial colonization is based on the biohalogenation of signal substances that interfere with bacterial communication. Biohalogenation is catalyzed by haloperoxidases, a class of metal-dependent enzymes whose activity can be mimicked by ceria nanoparticles. Transparent CeO_2_/polycarbonate surfaces that prevent adhesion, proliferation, and spread of *Pseudomonas aeruginosa* PA14 were manufactured. Large amounts of monodisperse CeO_2_ nanoparticles were synthesized in segmented flow using a high-throughput microfluidic benchtop system using water/benzyl alcohol mixtures and oleylamine as capping agent. This reduced the reaction time for nanoceria by more than one order of magnitude compared to conventional batch methods. Ceria nanoparticles prepared by segmented flow showed high catalytic activity in halogenation reactions, which makes them highly efficient functional mimics of haloperoxidase enzymes. Haloperoxidases are used in nature by macroalgae to prevent formation of biofilms via halogenation of signaling compounds that interfere with bacterial cell–cell communication (“quorum sensing”). CeO_2_/polycarbonate nanocomposites were prepared by dip-coating plasma-treated polycarbonate panels in CeO_2_ dispersions. These showed a reduction in bacterial biofilm formation of up to 85% using *P. aeruginosa* PA14 as model organism. Besides biofilm formation, also the production of the virulence factor pyocyanin in is under control of the entire quorum sensing systems *P. aeruginosa*. CeO_2_/PC showed a decrease of up to 55% in pyocyanin production, whereas no effect on bacterial growth in liquid culture was observed. This indicates that CeO_2_ nanoparticles affect quorum sensing and inhibit biofilm formation in a non-biocidal manner.

## Introduction

Biofilms are an accumulation of microorganisms, mostly bacteria, living in close community within a mucous substance^1^. They can form on almost any surface. Typical examples are plastic surfaces in the domestic environment (e.g., shower curtains, touch surfaces)^2^, or on consumer electronics^3^. Many industries suffer from the ill-effects of biofilm growth, which leads to costs for cleaning and maintenance.

Biofouling, i.e., biofilm adhesion, is mediated in the first by a protein layer, serving as a platform for cell attachment and subsequent bacterial colonization. There are several strategies to prevent biofilm attachment. The first is to use biocides, antibacterial agents that kill bacteria or other microorganisms. The second strategy is to repel the proteins and subsequently the cells, thus preventing biofouling. The third is to make self-cleaning surfaces like the lotus leaf^4^ where bacteria do not stick. While nature often uses a combination of these approaches, chemistry has focused either on antimicrobial coatings^5^ or on surface modification strategies (e.g., with polyethylene glycol (PEG)^6^, zwitterionic^7^ or amphiphilic polymers^6,8^, stimuli-responsive materials^9^, or by micro-nano structuring^10^, photoactivation^11^, or superamphiphobic/oleophobic surfaces)^12^ to prevent bacterial adhesion. Each of these methods has its limitations. Polymer coated surfaces suffer from mechanical weakness, poor substrate adhesion, non-uniform coating or insufficient long-term stability or cost–benefit ratio. Shortcomings of antimicrobial coatings are leaching of toxins and low biocompatibility. A simple, sustainable, and inexpensive method to prepare durable, polymer surfaces with uncompromised mechanical properties, which repel a variety of contaminants, has immediate relevance in many applications that suffer from the above issues.

A new and environmentally benign solution is inspired by nature. Macroalgae have evolved molecular mechanisms to limit biofilm formation (biofouling) on their surfaces^13^. The lack of microbial fouling of these algae is associated with the formation of brominated furanones that inhibit bacterial quorum sensing^32^, a process by which bacteria monitor cell density and regulate their communal behavior such as biofilm formation, virulence, or swarming motility among other responses^14–16^. Many bacteria secrete small signaling molecules, called autoinducers (AI)^14^. The halogenated messenger compounds have been shown to disrupt quorum sensing by displacing the signaling molecules from their corresponding receptor or by covalent modification, i.e. inactivation of the associated enzyme^14–16^.

These biohalogenation reactions are catalyzed by haloperoxidases (HPOs), a group of enzymes that use hydrogen peroxide, generated in micromolar quantities in sunlight, and halides (Cl^-^, Br^-^) to form hypohalous acids (HOX, X = Cl, Br). These short-lived reaction intermediates are powerful halogenating and oxidizing agents that halogenate a wide range of substrates^17–19^. Some halogenated metabolites have antimicrobial activity and/or prevent the formation of biofilms, i.e., nature uses oxidative halogenation as a chemical defense strategy against biofouling.

Several metal complexes^20–23^, V_2_O_5_^24^, and CeO_2_^25,26^ nanoparticles (NPs) can mimic the catalytic activity of haloperoxidase enzymes under ambient conditions^27^. Therefore, these NP enzyme mimics^28–31^ display a sustained and long-term activity at ambient conditions to produce sufficient amounts of hypohalous acids to prevent biofouling in moist environments or surface water films^32^. Incorporating these nanomaterials in polymers or coatings may reduce biofilm formation and lead to materials with long‐term stability. Assuming that concentrations of halide anions and peroxide on polymer surfaces are similar as in natural waters, a related behavior might be expected on polymer coatings^33,34^. This may open new and sustainable applications in everyday life (e.g., textiles, touch surfaces, food packaging, foils) and circumvent the use of biocides like silver or copper ions, antibiotics, chlorohexidine, or quaternary ammonium salts^35,36^.

Nanoceria is biologically benign^37–39^. Its application in transparent polymer coatings or nanocomposites, however, requires a large-scale synthesis of non-aggregated CeO_2_ NPs with well-defined size and crystallinity to enable blending or dispersing NPs in polymers with high optical transparency while maintaining antibacterial properties. Large scale routes to nanoceria are based on high-temperature gas phase reactions, which lead to highly agglomerated particles incompatible with the required transmittance and low haze in display films, coatings or lenses.

Precipitation in batch reactors is a straightforward route to ceria NPs, but it suffers from long cycle times and difficulties in controlling particle size due to poor heat and mass transfer. Alternative routes to CeO_2_ NPs are sol–gel or hydrothermal/solvothermal synthesis^40^. The size and morphology of CeO_2_ NPs can be tailored by adjusting the process parameters (e.g. pH value, reaction time, counter ions and addition of surfactants)^41–45^. Larger quantities of NPs (e.g. ceria, yttria, and zirconia nanoceramics) with low polydispersity and control over particle size can be obtained by continuous flow synthesis^46–50^. However, these reactions require high energy input, special equipment and they are difficult to parallelize.

Here we present a simple and highly scalable microfluidic benchtop system for high-throughput synthesis of CeO_2_ NPs with homogeneous size, morphology and crystallinity in segmented flow. The synthesis was carried out with oleylamine and cerium(III)-acetate in benzyl alcohol as reaction and water as carrier medium. Investigation of their surface chemistry revealed a very high haloperoxidase-like activity of the CeO_2_ NPs. Surface coating of polycarbonate panels with CeO_2_ NPs resulted in transparent nanocomposites with very low surface roughness and an enhanced Young's modulus as a measure of stiffness. CeO_2_/PC nanocomposites inhibit bacterial biofilm formation of *P. aeruginosa.* Production of the virulence factor pyocyanin in *P. aeruginosa* showed that the bacteria repelling properties are probably due to *quorum quenching*, an effect also termed *quorum quenching.* CeO_2_/PC nanocomposites may find application as biocide-free “green” antimicrobials on plastics for electronic, automotive, aircraft, or railway components, for drinking bottles, glasses and food containers, where traditional biocides are strictly prohibited because of toxicity. In the health sector this may prevent infections that are becoming increasingly troublesome with the emergence of drug resistant bacteria and help reducing the use of antibiotics.

## Results and discussion

### Batch synthesis

A single step approach was devised for the synthesis of ceria NPs based on a non-hydrolytic sol–gel route in benzyl alcohol. It allows excellent control over particle size, phase, and crystallinity, but also simplifies transfer into the flow setup^51–53^ Ce(ac)_3_ or Ce(acac)_3_ were used as starting compounds because of their availability and good solubility in benzyl alcohol. In a typical non-hydrolytic sol–gel reaction, the Ce(ac)_3_ precursor—or secondary complexes—experience an increased thermodynamic driving force to form “monomers”. Further reaction of these monomers with benzyl alcohol^51^ leads to the formation of CeO_2_ nuclei. The nucleation of nascent NPs is triggered by heating, and continued heating allows the nuclei to grow into mature NPs. Heating involves specific subtleties resulting from the interplay of reaction variables such as precursor concentration, solvent, reaction temperature and time. All these variables affect the final size and size distribution of the NPs.

A possible scenario could be as follows: In the reaction of cerium acetate hydrate (Ce(ac)_3_ ∙ × H_2_O) in benzyl ether an acid–base equilibrium ac^−^ + HOCH_2_C_6_H_5_ = ac–OCH_2_C_6_H_5_ + OH^− ^→ precedes the formation of Ce(OH)_3_ in an initial step at 120 °C (from Ce^3+^ and OH^−^ anions formed in the acid–base equilibrium). Ce(OH)_3_ is sensitive to aerial oxygen and oxidized to CeO_2_ in a topotactic condensation reaction, where water is eliminated^54^. In benzyl alcohol this water may be involved in a second acid–base reaction with oleylamine according to H_2_O + H_2_N-R = OH^−^ + ^+^NH_3_-R. In a competing reaction oleylamine acts as a capping agent in the surface complexation of the growing CeO_2_ NPs. The hexagonal structure of the Ce(OH)_3_ intermediate allows topotactic growth of anisotropic particles (e.g., nanorods) of CeO_2_ (which adopts the cubic fluorite structure)^55^. The capping agent plays an important role for the size distribution of the NPs by binding to the growth species and controlling its growth rate^56–58^. Terminating the reaction at this stage leads to the formation of single-domain crystalline CeO_2_ NPs with diameters of 2–7 nm, which could be dispersed in nonpolar solvents such as cyclohexane or toluene.

**Figure 1:**
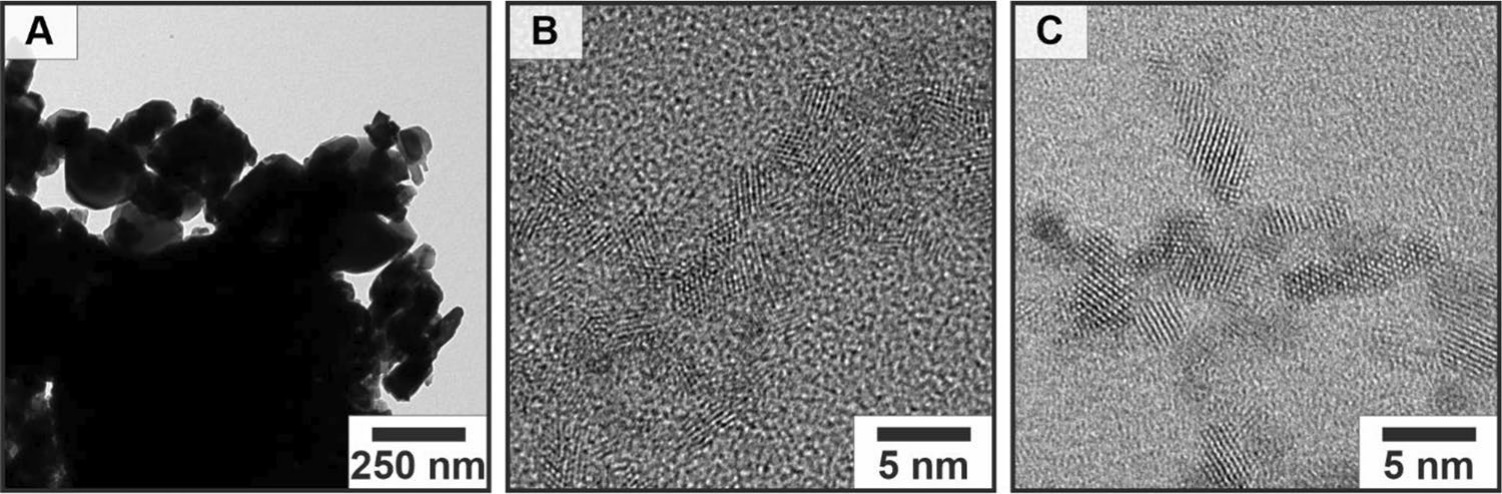
(**A**) TEM image of commercial CeO_2_. HRTEM images of CeO_2_ NPs obtained by non-aqueous sol–gel chemistry. (**B**) NPs obtained from batch chemistry and (**C**) under segmented flow conditions. Commercial CeO_2_ is produced by a gas-phase condensation process in which particles that are still hot are collected in a container. In this process, the hot particles sinter together and form large agglomerates, which do not form stable dispersions of isolated particles. In addition, no monodisperse particles are obtained in this type of mass production. Dispersions of polydisperse particles and large aggregates show strong light scattering and lead to cloudy opaque films after embedding in polymers.

Figure 1A shows commercially available CeO_2_ NPs synthesized on an industrial scale. They have an ill-defined morphology and strong agglomeration. For the latter application small NPs with a high surface area are necessary, therefore the control of the morphology and size is mandatory. CeO_2_ NPs from the batch (high-resolution transmission electron microscopy (HR)TEM images in Fig. 1B) are much smaller (two orders of magnitude) than their commercial counterparts and therefore suitable for application in nanocomposites. In the second step, the batch synthesis was transferred into a continuous setup.

### Synthesis of CeO_2_ NPs under segmented flow conditions

The HRTEM micrograph in Fig. 1C shows that the CeO_2_ NPs by segmented flow synthesis have essentially the same morphology as those obtained from the batch reaction (Fig. 1B). Compared to the commercial CeO_2_ particles (Fig. 1A) the NPs from the segmented flow synthesis show a uniform morphology and size.

However, clogging of the reactor could not be prevented under continuous flow conditions. Therefore, the synthesis was carried out under segmented flow using water as carrier medium. Advantages of this setup are the low costs and the boiling temperature of water. Water evaporates when the flow passes through the heating element. The water vapor leads to additional pressure between the segmented droplets of the reaction medium and thus prevents—independent of the flow rate—clogging of the reaction channel. A constant flow rate of the reaction medium and the water through the PTFE tube was achieved with a peristaltic pump. Nucleation was triggered by warming the reactant stream in a heating element.

Two heating elements (120° and 180 °C) were used to simulate the temperature profile of the batch process. The setup is shown in Figure S1A. The suitability of the PTFE tubing for the thermally induced formation of CeO_2_ NPs was assessed in preliminary experiments under continuous flow conditions. The residence times in each oil bath was set to 2.7 min to simulate the reaction conditions from the batch synthesis (vide supra). The reaction solution had a reddish color and turned purple after passing through the tubes. Two phases were obtained: the upper phase was an emulsion of water, benzyl alcohol and oleylamine, the lower phase contained the precipitated yellow NPs (Figure S1B).

**Figure 2:**
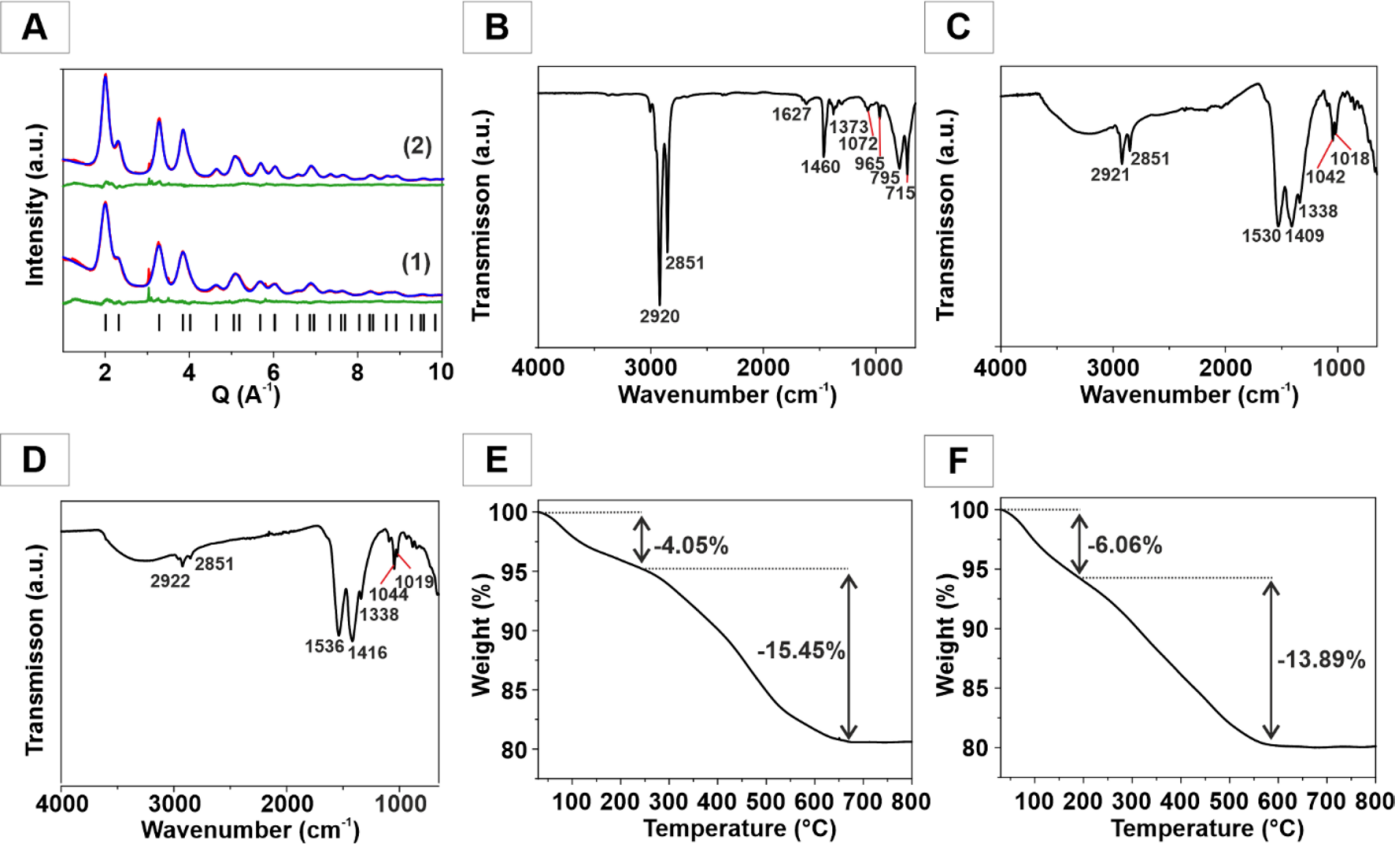
(**A**) X-ray powder diffraction patterns with Rietveld refinements of CeO_2_ NPs obtained by batch synthesis (1) and under segmented flow conditions (2). Experimental data (red line), Rietveld refinement (blue line), difference curve (green line). Ticks indicate theoretical positions of Bragg intensities. FT-IR spectra of (**B**) pure oleylamine, (**C**) CeO_2_ NPs obtained from batch chemistry, and (**D**) under segmented flow conditions. TGA traces of CeO_2_ NPs obtained under (**E**) batch and (**F**) segmented flow conditions.

Importantly, the reactions could be carried out in automated form with much shorter reaction times. For the batch synthesis, the product yield was 0.7 mg/min, while 15.8 mg/min (i.e., 22 times as much) were produced at the same temperature in segmented flow (Figure S1C). The average crystallite sizes of the NPs (bulk sample) were determined by powder X-ray diffraction (PXRD, Fig. 2 red line) and Rietveld refinement (blue line, difference green line)^59,60^. Details concerning the refinement are given in the Supporting Information (Table S1-2). The NPs are single-crystalline. The X-ray diffractograms of the CeO_2_ NPs by batch synthesis (Fig. 2A1) are very similar to those obtained by segmented flow reaction (Fig. 2A2) and show the expected intensities for (bulk) CeO_2_ (lattice parameter *a* = 5.42 Å). Reflection broadening allowed extracting the particle size (average 3.2 nm and 5.8 nm by Rietveld analysis of the PXRD patterns for CeO_2_ NPs (batch/segmented flow synthesis). The morphologies of the NPs from both processes are similar, NPs from the segmented flow process are slightly larger than those from the batch synthesis. Scale up was possible, due to the segmented flow setup, which avoided clogging of the tubes, i.e., it allows a high-throughput automated synthesis of single crystalline CeO_2_ NPs in large quantities.

### Surface chemistry of CeO_2_ NPs

The oleylamine surfactant required an analysis of the CeO_2_ surface chemistry to assess the effect of the organic ligands on the catalytic activity. FT-IR spectroscopy was used to identify the surface ligands of the as-synthesized CeO_2_ NPs. Figure 2B shows the spectrum of pure oleylamine. The spectra of the CeO_2_ NPs from the batch (Fig. 2C) and the segmented flow synthesis (Fig. 2D) indicate the presence of surface-bound oleylamine. The broad band between 3500 cm^−1^ and 3000 cm^−1^ envelopes the symmetric and asymmetric O–H stretches. The bands around 2920 cm^−1^ and 2850 cm^−1^ are characteristic for the C–H stretch of the terminal CH_3_ group^56^. The absorptions in the range of 1550 cm^−1^ to 1400 cm^−1^ and in the range from 1250 cm^−1^ and 1400 cm^−1^ and close to 1000 cm^−1^ are typical for the stretching and bending vibrations surface-bound bi- and polydentate carbonate groups^61^. The surfactant coverage was determined by TGA (Fig. 2E,F).

Figure 2E shows thermogravimetric traces of the CeO_2_ NPs from the batch synthesis. The first step in the diagram is associated with a mass due to surface-bound H_2_O (∆m_rel_ = 4.05%). This step is initiated between 50 °C and 200 °C. Between 200 °C and 650 °C there is another mass drop corresponding to the remaining organic residues and traces of carbonate being stripped off (∆m_rel_ = 15.45%). The remaining solid material was CeO_2_. NPs obtained under segmented flow (Fig. 2F) contained a higher amount of surface-bound H_2_O (∆m_rel_ = 6.06%) because they were prepared in aqueous environment, while the mass loss of organic residues and carbonate was lower (∆m_rel_ = 13.89%) compared to the batch synthesis. The FT-IR spectra of the products obtained by batch and segmented flow synthesis (Fig. 2C,D) show a weaker intensity of the vibration at around 2920 cm^−1^ and 2850 cm^−1^ for the C–H stretch of the terminal CH_3_ group. This indicates a lower share of surface-bound oleylamine. Consequently, a larger amount of H_2_O was bound onto the surface. Based on the spectroscopic and thermoanalytical data a temperature-dependent (185 °C, 500 °C, 800 °C) analysis of surface chemistry and the resulting catalytic activity of the NPs was carried out.

**Figure 3:**
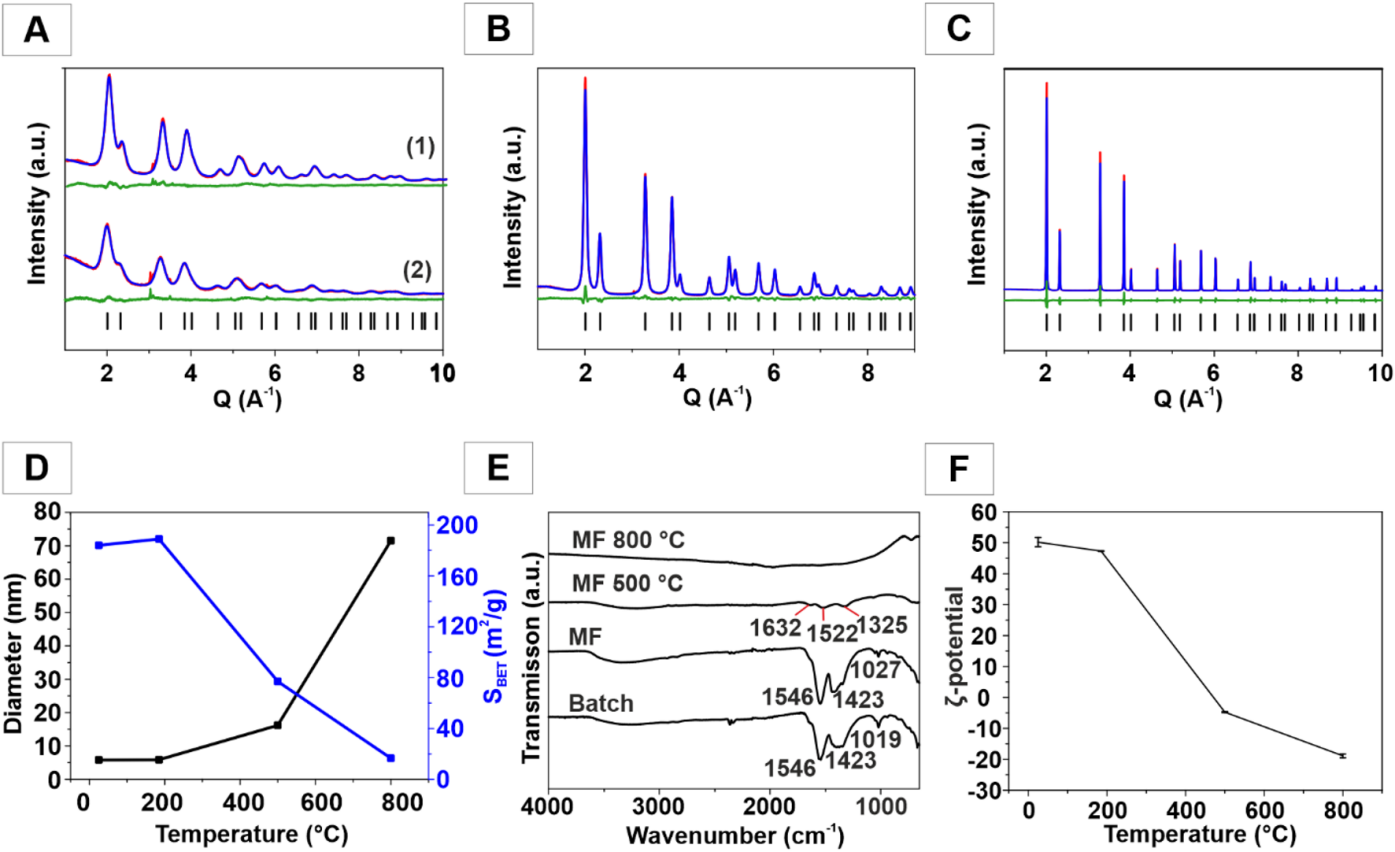
X-ray powder diffraction patterns with Rietveld refinements of CeO_2_ NPs obtained by batch synthesis (**A1**) and under segmented flow conditions (**A2**) after annealing for 5 h at 185 °C. (**B**) CeO_2_ annealed for 5 h at 500 °C and (**C**) 800 °C. Experimental data, Rietveld refinement and difference curve are shown as red, blue and green lines. Ticks indicate calculated positions of the Bragg intensities. (**D**) Average diameter and surface area as a function of annealing temperature. (**E**) FT-IR spectra of as-synthesized CeO_2_ NPs for different annealing temperatures and (**F**) ζ-potential as a function of annealing temperature.

X-ray analysis of the CeO_2_ NPs from the batch preparation (Fig. 3A1) and segmented flow synthesis after annealing for 5 h at 185 °C (Fig. 3A2) showed no significant changes compared to their non-annealed counterparts (Fig. 2A). CeO_2_ NPs from segmented flow synthesis were also annealed for 5 h at 500 °C and 800 °C. The NP diameter increased from 5.8 nm (185 °C) to 16.2 nm (500 °C) and finally to 71.5 nm (800 °C). The sharp reflections at 500 °C and 800 °C (Fig. 3B,C) and the HRTEM images (Figure S2A-C) show the increase in particle size. Since particle diameter correlated with surface area in an inverse fashion, the surface area was reduced at high annealing temperatures (Fig. 3D). The surface area for the NPs prepared at ambient temperature was 184 m^2^ g^−1^. It increased to 190 m^2^ g^−1^ after annealing at 185 °C. Surface ligands of the CeO_2_ NPs (ambient temperature) may cause errors in the BET measurements. Therefore, the surface area increased—formally—slightly after annealing, even though it is unfeasible physically. The surface areas decreased at higher temperatures to 78 m^2^ g^−1^ (500 °C) and 17 m^2^ g^−1^ (800 °C) due to grain growth.

The FT-IR spectra of the CeO_2_ NPs (batch preparation and segmented flow synthesis) showed after annealing for 5 h at 185 °C (Fig. 3E) no organic ligand anymore, indicated by the absence of the bands around 2920 cm^−1^ and 2850 cm^−1^, which are characteristic for the C–H stretch of the terminal CH_3_ group^56^. The absorptions at 1570 cm^−1^, between 1250 cm^−1^ and 1400 cm^−1^ and close to 1000 cm^−1^ are typical for the stretching and bending vibrations of surface-bound bi- and polydentate carbonate groups^61–63^. CeO_2_ shows acid/base properties. Its acidic behavior is responsible for OAM surface binding. Its base properties are responsible for the binding of carbonate groups. This binding of carbonaceous species is responsible for the use of CeO_2_ in gas-phase heterogeneous catalysis (e.g. in exhaust catalysts)^64,65^. It is also an important feature of hydroxylated and non-hydroxylated CeO_2_ NPs in aqueous environment, where active surface sites are blocked by adsorbed species.

After calcination for 5 h at 800 °C all vibrational bands of the surface species vanished (Fig. 3E), and the ζ-potentials of the NPs before and after annealing changed from positive to negative values. For CeO_2_ NPs (ambient temperature, segmented flow) an average value of 50.2 mV ± 1.5 mV was obtained. After annealing for 5 h at 185 °C the ζ-potential decreased slightly to 47.3 mV ± 0.12 mV. Annealing for 5 h at 500 °C reduced the ζ-potential to − 4.78 mV ± 0.17 mV, and after annealing for 5 h at 800 °C it was − 18.9 mV ± 0.59 mV (Fig. 3F).

### Haloperoxidase activity

**Figure 4:**
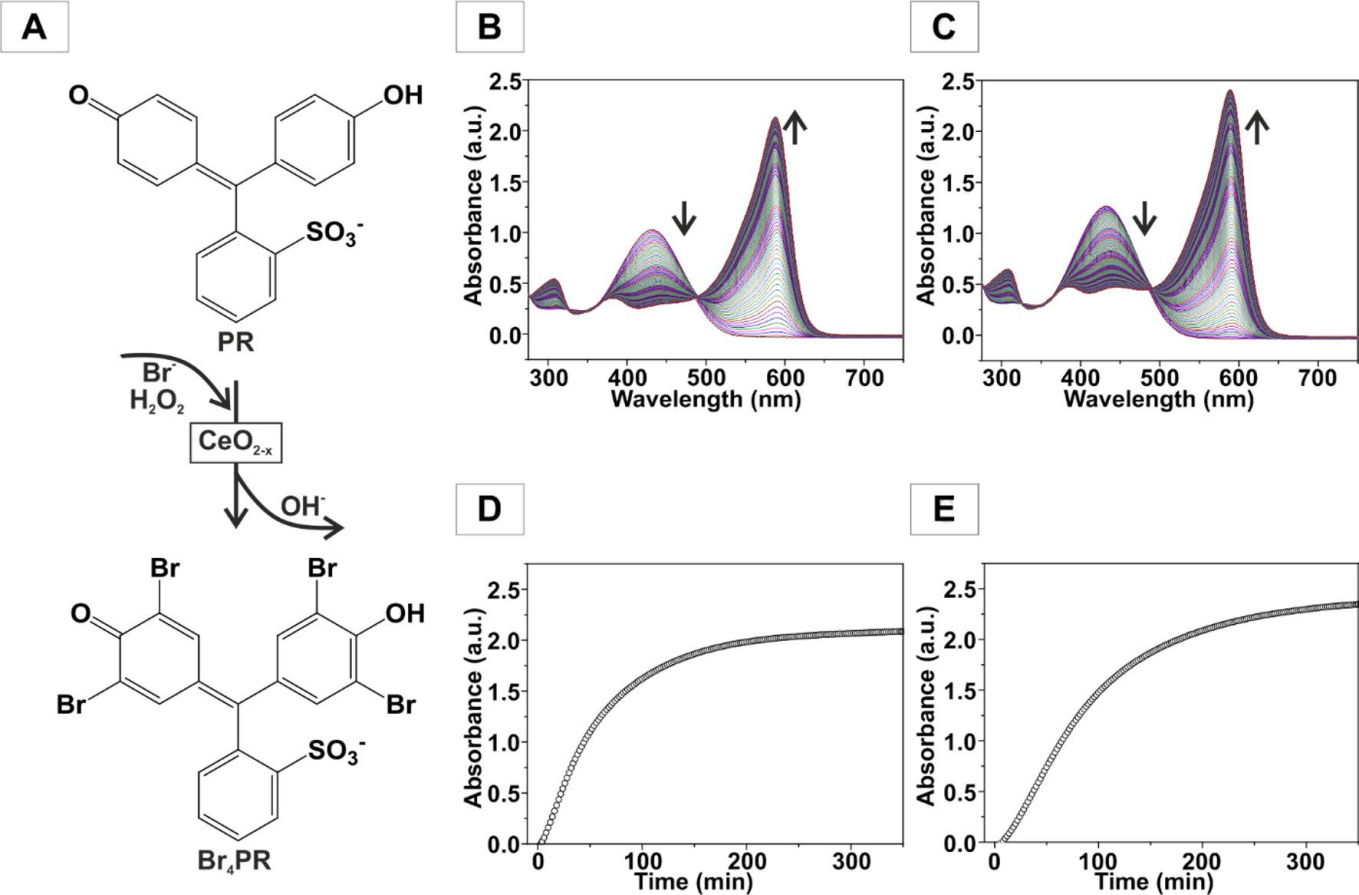
Scheme of the (**A**) oxidative bromination of PR. Spectral changes induced by the oxidative bromination of PR with time for the determination of the haloperoxidase activity of CeO_2_ obtained (**B**) from batch chemistry and (**C**) under segmented flow conditions after annealing at 185 °C, (**D**) the corresponding absorbance change at 590 nm as a function of time (**E**) the corresponding absorbance change. Assay components: CeO_2_ catalyst (25 µg/mL), H_2_O_2_ (300 µM), PR.

The haloperoxidase activity of CeO_2_ NPs was determined by UV/Vis spectroscopy with the phenol red (PR) assay (Fig. 4A). The spectra in Fig. 4B,C show the time-dependent intensity change of the two principal absorption bands for PR for CeO_2_ NPs (batch or segmented flow synthesis, after annealing at 185 °C) as haloperoxidation catalyst. The spectral changes and the slope of the absorbance correspond to the reaction rate. Figure 4D,E show the absorbance change for PR at 590 nm measured as a function of time for CeO_2_ (catalyst, 25 µg/mL), H_2_O_2_ (300 µM), PR (50 µM) and KBr (25 mM). The shift of the absorption bands is due to the fourfold oxidative bromination of PR to tetrabromophenol blue (TBPB).

As the haloperoxidase activity depends on the concentration of the H_2_O_2_ substrate, the Michaelis–Menten kinetics was evaluated by varying the H_2_O_2_ concentration. The kinetic data were fitted using the Hill equation. Table 1 shows the parameters derived from a fit to the Hill equation (Figure S3). Evaluating the kinetic parameters for CeO_2_ NPs obtained from the segmented flow synthesis (ambient temperature) was not possible, because their water dispersibility was low due to the OAM surface ligands.

**Table 1:** Kinetic parameters with H_2_O_2_ as substrate derived from the Michaelis–Menten equation.

Sample	v_max_ (µM min^−1^)	K_m_ (µM)	n
CeO_2_–185 °C	0.15	470.0	1.47
CeO_2_–500 °C	0.36	296.9	1.15
CeO_2_–800 °C	0.03	575.8	0.82

Brunauer–Emmett–Teller (BET) surface areas were determined to normalize the reaction rates to surface areas between 190 m^2^ g^−1^ (ambient temperature) and 17 m^2^ g^−1^ (800 °C). Table 1 shows that the bromination rate increases with the annealing temperature (185 °C vs. 500 °C) for CeO_2_. Based on the surface areas (190 m^2^g^−1^ vs. 78 m^2^ g^−1^) and the ζ-potentials (47.3 mV ± 0.12 mV vs. − 4.78 mV ± 0.17 mV) one may expect a higher activity for the sample treated at 185 °C. However, the bromination rate appears to depend not only on surface area and ζ-potential. Another factor might be the carbonate surface concentration of the CeO_2_ sample annealed at 185 °C, as surface bound CO_3_^2−^ groups may quench the reaction or block active sites. After annealing at 800 °C the bromination rate dropped significantly. The saturation rate (K_m_ value) increased, i.e., the affinity for H_2_O_2_ decreased. The Hill coefficient decreased initially from 185 to 800 °C. Thus, cooperative effects decrease by increasing the annealing temperature from 185 to 500 °C and finally to 800 °C. The catalytic activity described in terms of the turnover frequency (TOF) or the catalytic constant (k_cat_)^66^ depends on the catalyst surface area, time, and mass concentration. The rate of reaction (ROR) is defined by Eq. (1).

1 $$\mathrm{TOF}= \frac{{\mathrm{v}}_{\mathrm{max}}}{{\left[\mathrm{Cat}\right]}_{0}} \stackrel{{\left[\mathrm{Cat}\right]}_{0}\to {\mathrm{S}}_{\mathrm{BET}}\cdot\upbeta (\mathrm{Cat})}{\to }\mathrm{ROR}= \frac{{\mathrm{v}}_{\mathrm{max}}}{{\mathrm{S}}_{\mathrm{BET}}\cdot\upbeta (\mathrm{Cat})}$$

Here, the accessible surface area per mass equivalent of the catalyst is expressed through the BET surface (S_BET_ in m^2^ g^−1^). The turnover of PR to TBPB is surface specific because only the catalyst surface is accessible for the substrate. Equation (1) can be evaluated based on time (v_max_/M min^−1^), surface area (S_BET_/m^2^ g^−1^) and mass concentration (β(Cat)/g L^−1^) and yields the molar amount of TBPB that was formed by reaction of HOBr with PR per unit surface and time. The calculated ROR value for CeO_2_ (185 °C) is 0.031 µmol m^−2^ min^−1^, while CeO_2_ (500 °C) shows the highest ROR value (0.185 µmol m^−2^ min^−1^). For the CeO_2_ annealed at 800 °C we obtained a value of 0.071 µmol m^−2^ min^−1^. For the application of CeO_2_ NPs on polycarbonate surfaces there are two important requirements. (i) A high surface area to volume ratio is essential for catalytic activity. (ii) High dispersibility of the NPs is necessary to ensure uniform distribution of the NPs on the polycarbonate surface during the dip coating process. In this way, we could fabricate a smooth and transparent nanocomposite. CeO_2_ NPs annealed at 185 °C were the most successful candidate.

### CeO_2_/polycarbonate (PC) nanocomposites

**Figure 5:**
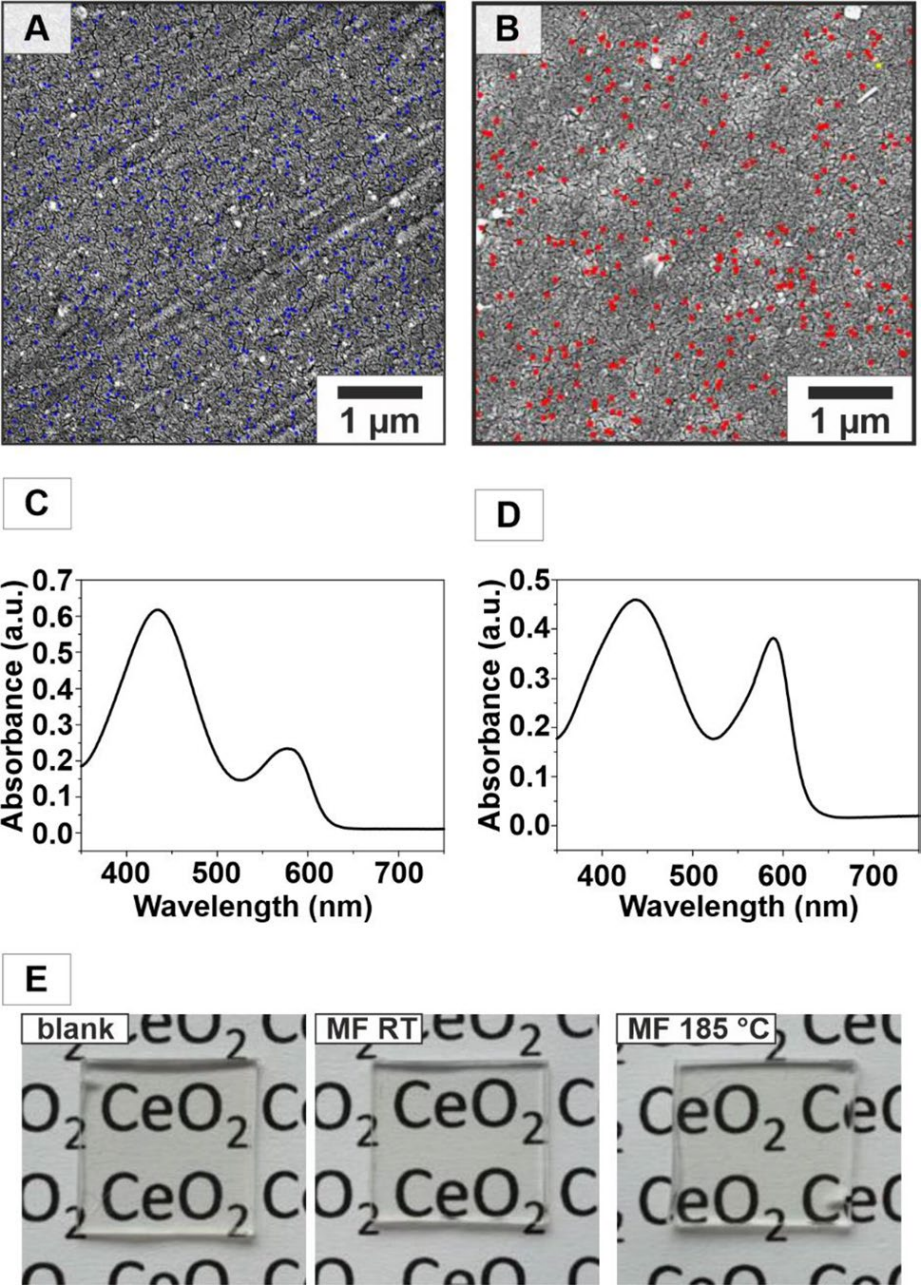
CeO_2_/polycarbonate panels. (**A**) SEM images of polycarbonate panels with CeO_2_ NPs from segmented flow synthesis with corresponding (EDX mapping marked as blue dots for Ce). (**B**) Polycarbonate panels with CeO_2_ NPs from segmented flow synthesis, annealed at 185 °C (EDX mapping marked as red dots). (**C**, **D**) Spectral changes induced by oxidative bromination of PR after 72 h of the CeO_2_/polycarbonate panels with (**C**) CeO_2_ NPs (not annealed) and (**D**) CeO_2_ NPs (annealed at 185 °C). (**E**) Image of a blank polycarbonate panel (left) polycarbonate panels with CeO_2_ NPs (not annealed/185 °C).

To bind a sufficiently large amount of CeO_2_ NPs to the PC surface, the PC panels were exposed to an oxygen plasma for 20 min. Contact angle measurements of polycarbonate with water, showed a decrease from 86.75° before treatment to 26.88° after oxygen plasma treatment (Figure S4), indicating a significant change in surface energy. The plasma treatment generated new oxygen containing groups such as C=O, O–C–C or O–C=O as reported by Shikova et al*.*^67^ Dip-coating of the plasma-treated PC panels in a dispersion of CeO_2_ particles in cyclohexane (ambient temperature) and water (185 °C) resulted in the binding of the particles to the polycarbonate surface by condensation reaction between the polar groups of polycarbonate surface and the free hydroxyl groups of the particles leading to a very dense and stable coating of CeO_2_ NPs on the PC surface (10 mg/mL, immersion speed of 60 mm/min, deposition time of 5 s and withdrawal speed 60 mm/min). The coated plates were dried in air (110 °C), excess CeO_2_ was removed by washing with ultra-pure MilliQ (QMQ) water, and the plates were dried at 80 °C overnight. Surface coating prevented embedding of CeO_2_ particles in the PC matrix without having an active surface area for catalytic reactions. It also circumvented the problem of producing stable dispersions from a mixture of CeO_2_ NPs and organic binders which often result in hazy films^68^ due to particle aggregation. The coating with CeO_2_ NPs was demonstrated by SEM/EDX (Fig. 5A,B, not annealed, annealed at 185 °C).

The binding of the CeO_2_ NPs to the surface proved successful, as both CeO_2_/PC panels showed high catalytic activity (demonstrated by conversion PR to TBPB in the PR assay, Fig. 5C,D). Importantly, coating of polycarbonate panels with CeO_2_ NPs did not lead to any color change. Highly transparent CeO_2_/PC sample panels were obtained, which showed no difference in transmission compared to uncoated PC panels (Fig. 5E; UV–vis absorption spectra in Figure S5).

Similar Young's moduli / hardness values were obtained for the CeO_2_/PC samples. The Young's moduli for CeO_2_/PC with annealed NPs slightly (3.74 GPa ± 0.06 GPa) increase compared to those of CeO_2_/PC with non-annealed NPs (3.73 GPa ± 0,12 GPa), while the hardness value slightly decreased for the former ones from 274.81 MPa ± 8.76 MPa (ambient temperature) to 265.80 MPa ± 9.86 MPa (annealed at 185 °C). These values are much higher than those which were obtained for pure PC 2.32 ± 0.02 GPa (2.4 GPa ISO 527-1,-2) and 178.77 MPa ± 2.20 MPa^69^. As expected, the Young's modulus increased with surface-bound CeO_2_ content. Comparative hardness values of polystyrene, polypropylene, polyethylene, poly(ethylene terephthalate), and poly(methyl methacrylate) polymer films without NPs coating are typically close to 10 MPa^70^, while the Young's modulus and hardness of pure ceramic CeO_2_ coatings prepared by magnetron sputtering are 80.5 ± 1.3 GPa and 4.1 ± 0.3 GPa^71^. Although Young´s modulus and scratch resistance are independent parameters, the ratio E/H of Young´s modulus and hardness corresponds to the ratio of the contact depth over the total penetration^72^. This indicates that CeO_2_ coating of PC panels increases the scratch resistance of polycarbonate surfaces.

### CeO_2_/PC nanocomposites inhibit biofilm formation and affect pyocyanin production in P. aeruginosa

CeO_2_/PC composites were tested on their potential to inhibit bacterial biofilm formation and on the ability to interfere with bacterial quorum sensing with *P. aeruginosa*, a Gram-negative soil bacterium, recognized for its ubiquity, its antibiotic resistance mechanisms, which is known to contaminate drinking water distribution systems. Furthermore, *P. aeruginosa* is a potent biofilm producer and therefore widely used as an indicator strain^73^. Additionally, *P. aeruginosa* is known to be responsible for opportunistic infections in situations with limited barriers, such as skin defects in wounds, or epithelial impairment in advanced stages of chronic pulmonary diseases or cystic fibrosis^74^.

**Figure 6:**
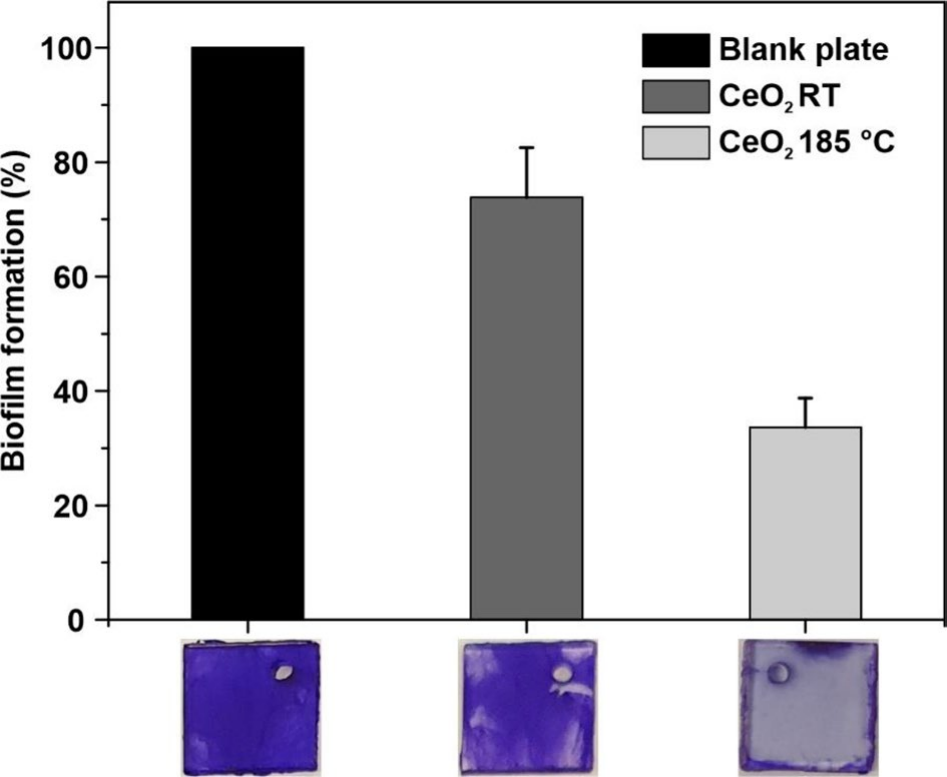
Bacterial biofilm formation on CeO_2_-coated PC surfaces. Crystal violet staining assay of *P. aeruginosa* PA14 grown in LB broth. The bacteria were cultivated aerobically for 72 h under gentle movement (150 rpm) at 30 °C on non-coated (blank plate) and CeO_2_-coated PC surfaces annealed at 185 °C or not annealed (RT). Then, the planktonic cells were removed, and the attached cells were stained with crystal violet. After solubilization of the bound crystal violet, the quantification was performed via measurement of absorbance in a plate reader at 575 nm. The stained plates are shown at the bottom of the figure. Error bars represent the standard deviation of three independently performed experiments.

To test biofilm formation on CeO_2_ coated PC surfaces, the bacteria were grown for 72 h at 30 °C on different composites placed in 24-well plates. After removing the planktonic cells, the attached bacteria were stained with crystal violet. In comparison to the uncoated PC plate, both nanoparticle composites were able to decrease bacterial biofilm formation (Fig. 6). CeO_2_ particles annealed at 185 °C resulted in a 65% reduction in of *P. aeruginosa* biofilm formation, showing a stronger effect than untreated CeO_2_ particles, where the reduction was only 30%. While the biofilm on the untreated PC plate was distributed equally all over the surface, the distribution was inhomogeneous on both CeO_2_ RT/PC composites and the CeO_2_ 185 °C/PC nanocomposites (Fig. 6).

**Figure 7:**
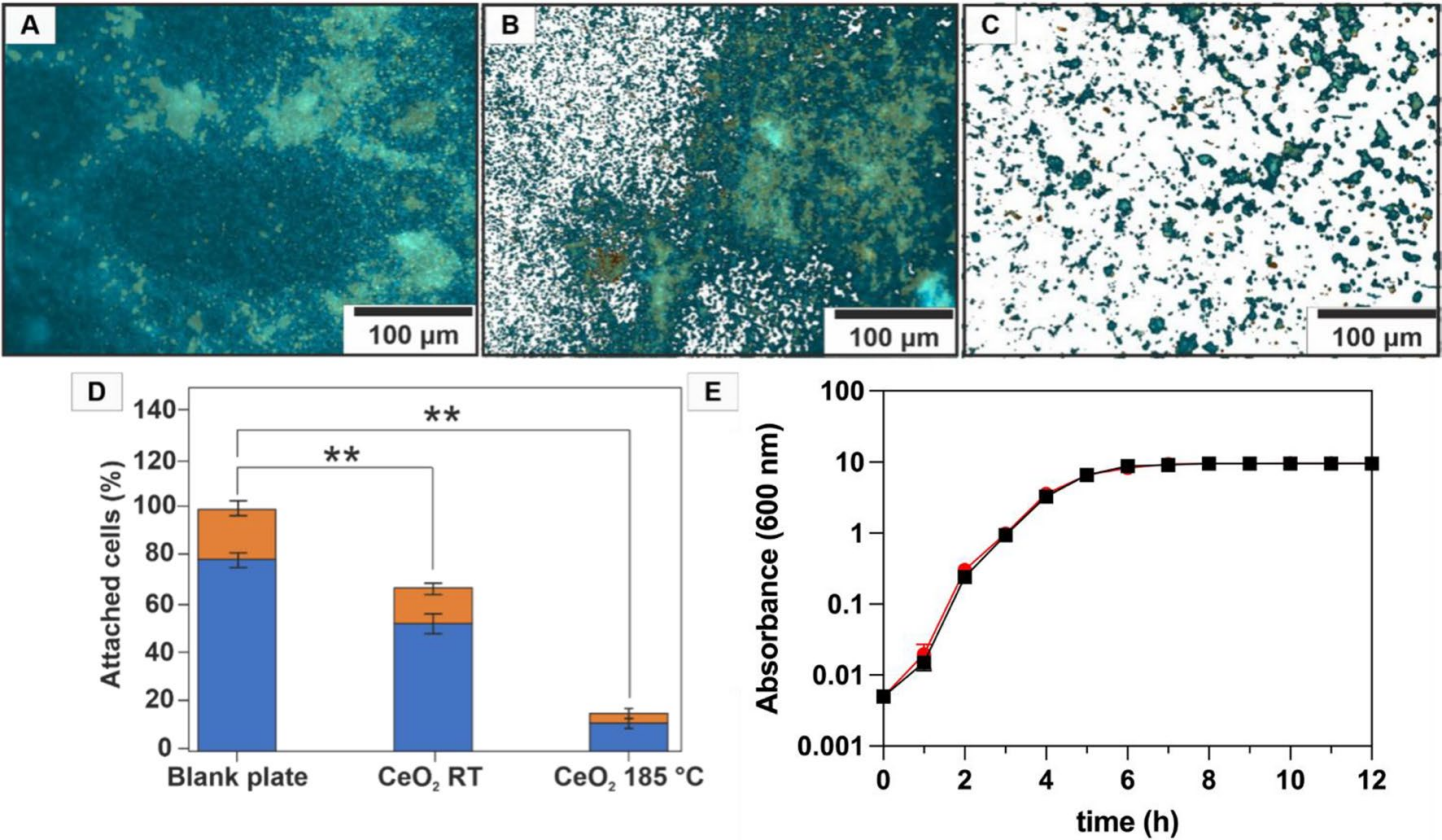
Effect of CeO_2_-coated PC surfaces on cell adhesion and growth of *P. aeruginosa* PA14. The bacteria were cultivated for 72 h under gentle shaking (150 rpm) at 30 °C on non-coated and coated CeO_2_/PC composites. The planktonic cells were removed, and the remaining bacteria attached to the surfaces were stained with SYTO 9 and propidium iodide for 30 min at 30 °C. The surfaces were then analyzed by epifluoresence surface microscopy. The pictures show bacterial attachment on (**A**) non-coated, on (**B**) CeO_2_ RT/PC and (**C**) CeO_2_ 185 °C/PC composites. The effect of the incorporated CeO_2_ NPs on bacterial biofilm formation was quantified by analyzing three different squares of 300 µm × 400 µm in size by quantifying the portion of green (SYTO 9) and red fluorescence color (propidium iodide) representing (**D**) total and live cells (blue) count and respectively dead cells (orange). The asterisks (**) indicate statistically significant differences with a *p*-value smaller than 0,001. (**E**) Growth curve of *P. aeruginosa* with (black) and without (red) addition of 2% (w/v) CeO_2_. Error bars represent standard deviation of three independently performed experiments.

Furthermore, we analyzed biofilm formation on CeO_2_ coated PC surfaces by quantifying single live and dead cells on the surfaces. For that purpose, the *P. aeruginosa* PA14 was grown for 72 h at 30 °C on the different PC samples placed in 24-well plates. After removing the planktonic cells, the attached bacteria were stained with SYTO 9 (total and live cell count) and propidium iodide (dead cell count). In comparison to the blank plate (Fig. 7A), both CeO_2_/PC composites showed a decrease in bacterial cell attachment. While the biofilm on the blank plate was equally distributed all over the surface, less biofilm clusters were visible on the CeO_2_ RT/PC (Fig. 7B) and much less as well as smaller clusters on the CeO_2_ 185 °C/PC (Fig. 7C) composites. Although both nanoparticle composites were able to reduce the bacterial attachment, resulting in a reduced number of cell clusters on the surface, the most promising effect could be achieved with the CeO_2_ 185 °C/PC composites. In comparison to the blank plate, approximately 60% of the cells were attached to the CeO_2_ RT/PC surfaces, and only 15% on the CeO_2_ 185 °C/PC composites (Fig. 7D). Furthermore, the composites had no effect on the life/dead cell-ratio, as the portion of dead cells on the treated surfaces were comparable to that on the blank plate (approximately 15–20%) (Fig. 7D). These data clearly show the inhibitory and non-biocidal effect of the composites on bacterial biofilm formation. This could be also demonstrated in liquid culture. The addition of CeO_2_ to a liquid culture of *P. aeruginosa* PA14 had no effect on the growth of the bacteria at 30 °C (Fig. 7E). Therefore, CeO_2_ must interfere with bacterial biofilm formation on another than a toxic way.

For the organization within a biofilm, bacteria usually communicate via small diffusible molecules sensing their cell count, a process called quorum sensing^75^. The CeO_2_ NPs promote the production of halogenated compounds, which are similar to quorum sensing molecules of Gram-negative bacteria. Therefore, we assumed that the non-toxic biofilm inhibiting effect of CeO_2_ composites might be due to a putative interference with the bacterial cell–cell communication, an effect that is generally referred to as quorum quenching^76^.

**Figure 8:**
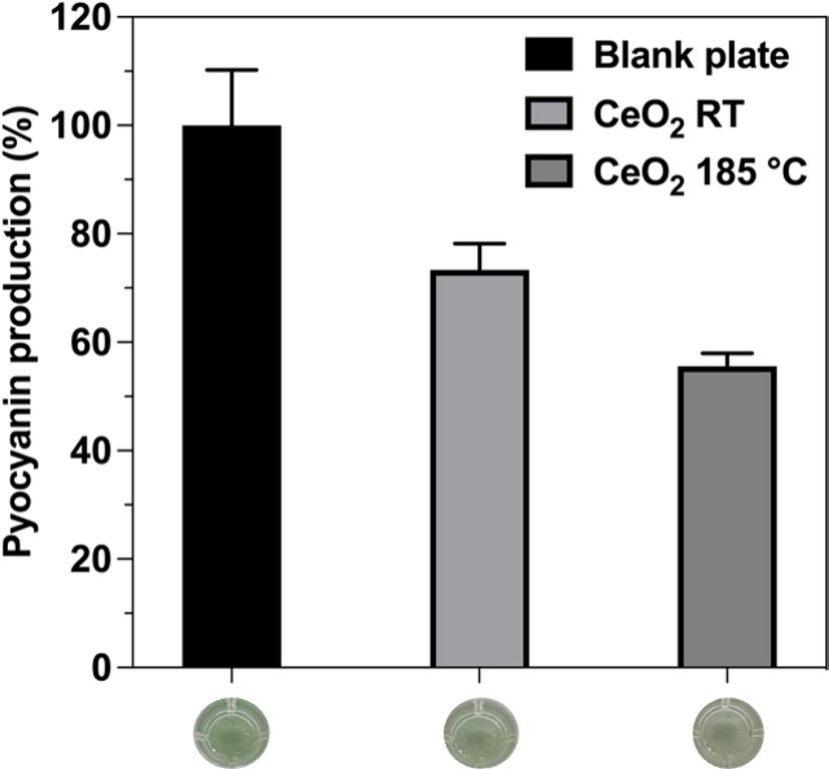
Effect of CeO_2_/PC nanocomposites on the pyocyanin production in *P. aeruginosa* PA14. The bacteria were cultivated for 72 h under shaking conditions (350 rpm) at 37 °C on non-coated and coated PC plates. The plates were then removed, and the remaining culture fluid was analyzed for pyocyanin occurrence in a plate reader at 695 nm. Error bars represent standard deviation of three independently performed experiments.

To get first evidence on the molecular mechanism of how CeO_2_/PC inhibit bacteria biofilm formation, we analyzed its effect on the production of the virulence factor pyocyanin in *P. aeruginosa* PA14. Besides biofilm formation, the biosynthesis of pyocyanin is also under control of the entire quorum sensing systems in these bacteria^77^. For that purpose, the bacteria were cultivated for 72 h at 37 °C on blank plates and on CeO_2_/PC composites. Then, the PC plates were removed, and the remaining culture fluid was analyzed on the presence of pyocyanin as described under *Materials and methods*. Both nanocomposites had a negative effect on pyocyanin production (Fig. 8). However, CeO_2_/PC composite annealed at 185 °C showed the highest decrease of up to 55% compared to the control cultures. Since *P. aeruginosa* is known to communicate via a distinct cell–cell signaling scheme^78^, it can be assumed that the NPs act on at least one of the three known quorum sensing systems. The results regarding inhibition of biofilm formation and pyocyanin production in *P. aeruginosa* finally showed the applicability of CeO_2_/PC nanocomposites to inhibit bacterial communication—most likely—and therefore bacterial biofilm formation as a non-toxic strategy.

## Conclusion

CeO_2_ NPs were coated on polymer surfaces to fabricate highly transparent and hard polymer/nanoparticle composites, which display the intrinsic haloperoxidase-like characteristics of the CeO_2_ NPs as demonstrated by the bromination of PR and the formation of the virulence factor pyocyanin, which is regulated by the quorum sensing system. Since *P. aeruginosa* is known to communicate via a distinct cell–cell signaling scheme, it can be assumed that the biomimetic activity of the CeO_2_ NPs acts on at least one of the three known quorum sensing systems. An effective segmented flow synthesis of CeO_2_ NPs was devised. It allows the continuous synthesis of non-agglomerated material with a single mixer to fabricate nanomaterials for polycarbonate displays in large quantities. The yield can easily be enhanced by parallelization. The highly reproducible (automated) synthesis of CeO_2_ NPs in copious amounts allowed to systematically analyze the role of particle size, ion concentration and surface chemistry on their (bio)catalytic properties.

An important point is that ceria does not leach because of its extremely low solubility (solubility product log K_L_ =  − 60)^79,80^. This is also the reason why cerium—despite its abundance (comparable to copper)^81^—is involved in biological processes only under extreme conditions in volcanic mudpots^82^. The low solubility makes ceria nanoparticles also highly biocompatible: little or no cytotoxic effects were observed (depending on the uptake pathway)^83^. Ceria nanoparticles have been proposed for biomedical applications (e.g. in sun creams)^54^, and it was demonstrated with in vivo experiments that ceria NPs can even protect cells against irradiation and oxidative stress^55,84^.

In contrast, various metal NPs (e.g., Cu or Ag) preventing biofouling are known to be harmful to the environment^85^. V_2_O_5_ NPs showed excellent performance to prevent biofouling on surfaces in marine environments^43^, but leaching leading to soluble polyvanadates in combination with reported carcinogenic and mutagenic effects make any practical application unlikely^86–89^. TiO_2_ and SnO_2_ nanoparticles show antibiofouling properties, but their mode of biocidal activity TiO_2_ is associated with the induction of oxidative stress due to photogenerated reactive oxygen species under UV irradiation. The production costs of TiO_2_ and SnO_2_ dispersible nanoparticles are moderate^90,91^. TiO_2_ particles can be incorporated into various polymers, such as polyamide^10^ poly(lactic-co-glycolic acid)^92^ or polytetrafluorethylene^93^ with antibiofouling properties. Titania nanoparticles have a broad activity spectrum against microorganisms, and they have been considered environmentally friendly with non-contact biocidal action^94^. Their recent classification as a “suspected carcinogen” by the EU, however, may lead to restrictions or even a ban on their chemical use in consumer products^95^. CeO_2_ offers a sustainable alternative to TiO_2_, with non-toxic properties and high abundance, more importantly catalytic activity does not depend on UV irradiation.

Because of their non-toxicity and environmental sustainability polycarbonate/CeO_2_ nanocomposites may find broad application as green antimicrobials on plastics for automotive, aircraft, or railway components, for drinking bottles, glasses and food containers, where traditional biocides (toxines, antibiotics, etc.) are strictly prohibited because of toxicity, non-target impacts or the evolution of resistance by micro-organisms. Other applications could be touch panels, sporting helmets, glasses, fountain pens, portable devices for consumer electronics like smart phones, audio player cases, computer cases, durable, lightweight luggage, musical instruments, and toys. Especially in the health sector this may prevent infections that are becoming increasingly troublesome with the emergence of drug resistant bacteria and reduce the use of antibiotics.

## Experimental details

### Materials

Ce(ac)_3_ ∙ × H_2_O (99,9% Sigma Aldrich), oleylamine (> 50% TCI Chemicals), benzyl alcohol (99% Acros Organics), deionized water, cyclohexane (99,8% Fisher Scientific), ethanol (98% Honeywell), H_2_O_2_ (35% Roth), fluorinated ethylene propylene tube with 1/16´´ OD and 1 mm ID (Fisher Scientific), peristaltic pump reglo ICC (Ismatec), Polycarbonate (thickness 1 mm, Goodfellow).

### Batch synthesis

Before the synthesis Ce(ac)_3_ ∙ × H_2_O was dried for 10 min at 130 °C in an oven. Subsequently 0.5 mmol (150 mg) of dried Ce(ac)_3_ was transferred into a 100 mL three-necked flask, furthermore 7 mL of benzyl alcohol and 3 ml of oleylamine were added. The solution was heated up to 120 °C (5 °C/min) and kept at the temperature for 20 min. Then it was heated up to 180 °C (5 °C/min) for 30 min with a stirring speed of 400 rpm. The reaction solution was subsequently cooled down to room temperature. For the purification the product was washed three times with ethanol and cyclohexane and dried under vacuum at room temperature.

### Segmented flow synthesis

Before the synthesis Ce(ac)_3_ ∙ × H_2_O was dried for 10 min at 130 °C in an oven. Subsequently, 1.4 mmol of dried Ce(ac)_3_ was transferred into a beaker, 21 mL of benzyl alcohol and 9 mL of oleylamine were added, and the solution was stirred at room temperature. Deionized water was added in a second beaker. The overall tube length was 9 m. The first segment (3 m) was put in an oil bath with a temperature of 120 °C, the second segment (3 m) was heated in an oil bath to 180 °C, the last segment (3 m) was kept at room temperature. The two solutions were pumped separately, the flow rate for the metal precursor solution was 2 mL/min, the flow rate for the deionized water was 1.5 mL/min. The two solutions were connected through a T-fitting. The nanoparticles were washed three times with ethanol and cylclohexane and dried under vacuum at room temperature. The reaction mixture was also scaled up to the tenfold and even 20-fold of the amount of the original batch synthesis.

### Powder X-ray diffraction

X-ray diffraction patterns were recorded on a STOE Stadi P diffractometer equipped with a Dectris Mythen 1 k detector in transmission mode using Mo Kα_1_ radiation. Crystalline phases were identified according to the PDF–2 database using Bruker AXS EVA 10.0 software^96^. Full pattern profile fits (Pawley/Rietveld) were performed with TOPAS Academic 6.0 applying the fundamental parameter approach^59,60^.

### Transmission electron microscopy

Transmission Electron Microscopy (TEM) mages for determining the size and morphology were acquired on a FEI Tecnai G2 Spirit microscope operating at 120 kV (LaB_6_ filament), equipped with a Gatan US1000 CCD-camera (16-bit, 2048 × 2048 pixels), using the Gatan Digital Micrograph software^97^. Samples for TEM were prepared by placing one drop (10 µL) of a diluted NP solution in chloroform (0.1 mg mL^−1^) on a carbon-coated copper grid and by letting it dry at room temperature. Size evaluation of individual nanoparticles on the TEM images was performed with ImageJ^98^.

### High resolution transmission electron microscopy

The Samples were prepared adding a drop of diluted Nanoparticles in Cylcohexan on a carbon coated copper grid. The samples were measured with the FEI Tecnai F30 ST TEM at 300 kV acceleration voltage equipped with a Gatan US4000 4 k CCD camera.

### Scanning electron microscopy

Scanning Electron Microscopy (SEM) images for demonstrating the presence of CeO_2_ on the surface of PC were acquired on a FEI Nova NanoSEM with a EDX-detector (EDAX-Pegasus X4M). The samples were coated with gold before investigation and observed at 15 kV.

### IR spectroscopy

ATR-IR spectra were measured on a Nicolet iS10 Spectrometer manufactured by Thermo Scientific. The spectra were recorded in a frequency range from 650 cm^−1^ to 4000 cm^−1^ with a resolution of 1.4 cm^−1^ per data point.

### Thermogravimetry (TGA)

The measurements were conducted using a Perkin Elmer TGA Pyris 6 (30 to 800 °C, heating rate of 5 °C·min^−1^, oxygen atmosphere, the sample weight was about 10 mg).

### Brunauer–Emmet–Teller (BET)

The specific surface area was determined using a *3P micro 300 adsorption–desorption device from 3P instruments*. Measurements were conducted with an appropriate amount of sample (150 mg) at 77 K with nitrogen as analysis gas.

### Preparation of CeO_2_/polycarbonate nanocomposites

Polycarbonate (PC) plates (ca. 1 × 1 cm) were washed with MQ-water and ultrasonicated for 10 min in isopropanol to remove contaminants. After that, PC plates were dried in air at 80 °C overnight. PC substrates were exposed to oxygen plasma for 20 min to produce hydroxyl groups onto the surface. The oxygen plasma was created at a pressure = 0.1 mbar and a power = 300 W. The pretreated PC plates were dip coated in a CeO_2_ dispersion (10 mg/mL) in cyclohexane with a dipping speed of 60 mm/min, deposition time of 5 s, and withdrawal speed of 60 mm/min. After dip coating, the coatings were dried in air at 110 °C for 16 h. Finally, CeO_2_-coated PC substrates were washed with MQ-water to remove excess CeO_2_ from the surface and dried at 80 °C overnight.

### Nanoindentation

Young’s modulus and hardness were measured by nanoindentation using a MFP Nanoindenter (Asylum Research, Santa Barbara, CA) equipped with a diamond Berkovich indenter. Each series of indentations was done on a grid of 2 × 6 indents on a 90 × 90 µm^2^ area at three different positions on the sample, i.e., 36 indents per series. The Young’s moduli and hardness were calculated by fitting the indentation curves according to the Oliver-Pharr method^99^ using the analysis software of the nanoindenter. The Young’s modulus was obtained as *elastic* response of the sample upon unloading, i.e., from the slope of the onset of the unloading curve. The hardness of the material was obtained as the ratio of maximum applied load divided by the indenter contact area at that load. It is indicative of the resistance of the material against *plastic* deformation.

### Contact angle measurements

The contact angle was measured with the contact angle measurement system OCA35 and the water drop was deposited by a Hamilton syringe (100 µL) with a hydrophobic needle.

### HPO activity measurements

The activity measurements were performed with the phenol red assay. Phenol red (PR) is converted to tetrabromophenol blue (TBPB) in the presence of a bromide source (KBr), H_2_O_2_ and CeO_2_ NPs dispersed in MilliQ water. The kinetic measurements were carried out on a Varian Cary 5G UV/Vis spectrometer in the wavelength range from 300 to 750 nm. For the measurements, CeO_2_ NPs (25 µg/mL) were dispersed in MilliQ water (18.2 MΩ cm^−1^), and H_2_O_2_ (300 µM), PR (50 µM), and KBr (25 mM) were added to the solution. The solution was stirred for 2 min after the addition of PR to obtain a homogenous solution. The baseline was measured with a mixture of NPs, KBr and PR dissolved in MilliQ water. H_2_O_2_ was added prior to the measurement.

### Michaelis menten kinetics

The Michaelis Menten kinetics were measured by adding different amounts H_2_O_2_ to a stirred solution of PR, KBr and the NPs. For this, standard solutions of KBr (250 mM) and PR (1 mM) were prepared separately by dissolving the appropriate amount in MQ-water under sonication. As next step a mixed standard solution of PR with KBr was prepared by dilution of the standard solutions. The final solution contained 50 µM PR and 25 mM KBr. From this solution 2 mL were mixed in a 2.5 mL quartz cuvette with 62.5 µL of a nanoparticle dispersion (1 mg/mL). The amount of H_2_O_2_ was introduced by adding always the same amount (50 µL) of an appropriate H_2_O_2_ dilution. For this purpose, a 100 mM H_2_O_2_ standard solution was prepared as a starting solution and the dilution was done immediately before the addition. Dilution was achieved by mixing MQ-water with the H_2_O_2_ initial solution (e.g., 95:5 v/v (water/H_2_O_2_(100 mM)) for a 5 mM H_2_O_2_) to prepare the diluted H_2_O_2_ solution. All kinetics were run for a reaction time of 6 min. The absorbance change was measured at a wavelength of λ = 590 nm. All data points were fitted linearly in an interval of 2–5 min, to achieve a linear relation between the data. The amount of TBPB was calculated from the Lambert–Beer law. The extinction coefficient was determined by measuring five TBPB solutions containing different amounts of dye (range c(dye) = 0.002–0.018 mM) in a quartz cuvette (d = 1 cm) to fit into the range of 0.1 < A_590_ < 1. An extinction coefficient of 47,111 L mol^−1^ cm^−1^ was determined for pure TBPB in water measured with a Varian Cary 5G spectrophotometer.

### Bacterial strains and growth conditions

*P. aeruginosa* PA14 was used for biofilm and growth assays, which is a clinical isolate with increased virulence compared to the reference laboratory strain PAO1 and is resistant against a large number of antibiotics)^100^. *P. aeruginosa* was aerobically cultivated in LB medium [1% (w/v) NaCl; 1% (w/v) tryptone; 0.5% (w/v) yeast extract] at 30 °C. For preparation of agar plates, 1.5% (w/v) agar was added to the medium. For monitoring growth curves *P. aeruginosa* PA14 cultures were grown overnight at 30 °C in LB medium. Overnight cultures were washed with sterile 0,85% NaCl (w/v) solution to remove residual media. Cultures were diluted in fresh LB medium to obtain equal optical densities (OD_600_) of 0,005. Growth of the cultures was assessed in LB broth in presence and absence of 2% CeO_2_ NP´s (w/v). Growth was monitored by determining absorbance at 600 nm using Ultrospec 2100 pro UV/Vis Spectrometer (Biochrom, Berlin, Germany) cuvette reader for 12 h at 30 °C. All experiments were performed in triplicate.

### Biofilm assays

For quantification of bacterial biofilm production, a modified method of previous published protocols was used^100^. Briefly, *P. aeruginosa* was aerobically cultivated in LB medium over night at 30 °C. The cultures were then diluted in LB in a volume of 1 ml per well of a 24-well polystyrene microtiter plate (Sarstedt, Nürnbrecht) at a final Optical Density at 600 nm (OD_600_) of 0.5. Additionally, KBr (Roth, Karlsruhe, Germany) and H_2_O_2_ (Roth, Karlsruhe, Germany) at a final concentration of 32 mM and 0.8 mM, respectively, were added to the wells. Then, the blank plates and CeO_2_/PC composites were subjected. Subsequently, the microtiter plate was incubated for 72 h under gentle shaking (150 rpm) at 30 °C. H_2_O_2_ was added stepwise at a final concentration of 0.8 mM every 24 h. Then, the plates were rinsed with water to remove the planktonic cells. After drying for 5 min, 1 ml of 1% (w/v) crystal violet (Merck, Darmstadt) was added to the wells containing the plates. After 30 min incubation at room temperature, unbound crystal violet was removed by gently submerging the plates for two times in water. The plate was then air-dried over-night at room temperature. For quantification, 1 ml of 30% (v/v) acetic acid (Roth, Karlsruhe) was added to the plates to solubilize the crystal violet from the biofilm. After 15 min of incubation at room temperature, absorbance was quantified in a plate reader (Tecan, Salzburg) at 575 nm.

### Pyocyanin analyses

For pyocyanin production, an alternate method of already published protocols were used^100^. *P. aeruginosa* was cultivated in LB medium over night at 30 °C. The cultures were then diluted in LB, in a volume of 1 ml per well of a 24-well polystyrene microtiter plate (Sarstedt, Nürnbrecht) at a final OD_600_ of 0.5. Additionally, KBr (Roth, Karlsruhe, Germany) and H_2_O_2_ (Roth, Karlsruhe, Germany) at a final concentration of 32 mM and 0.8 mM respectively were added to the wells. The non-coated plates and CeO_2_/PC composites were then added to the wells, respectively. The microtiter plate was then incubated for 72 h under shaking conditions (350 rpm) at 37 °C. Every 24 h H_2_O_2_ was added at a final concentration of 0.8 mM. Afterwards, the composites were taken out of the wells and the remaining supernatant inside the wells was collected in a microreaction tube (Eppendorf, Germany). Cells were separated from culture fluids via centrifugation at 16 × 1000 g for 15 min. Then, the supernatant was passed through 0.22 μm filters (Merck, Darmstadt). Cell-free culture fluids were then analysed for pyocyanin in a plate reader (Tecan, Salzburg) at 695 nm.

### Epifluorescence microscopy and fluorescence quantification

*P. aeruginosa* PA14 was cultivated in LB medium over night at 30 °C. The cultures were then diluted in LB, in a volume of 1 ml per well of a 24-well polystyrene microtiter plate (Sarstedt, Nürnbrecht) at a final OD_600_ of 0.5. Additionally, KBr (Roth, Karlsruhe, Germany) and H_2_O_2_ (Roth, Karlsruhe, Germany) at a final concentration of 32 mM and 0.8 mM, respectively, were added to the wells. The non-coated blank plates and nanoparticle composites were then added to the wells. The microtiter plate was then incubated for 72 h under gentle shaking (150 rpm) at 30 °C. Every 24 h H_2_O_2_ was added at a final concentration of 0.8 mM. Then, the plates were rinsed with water to remove the planktonic cells. Afterwards, the plates were placed in 1 ml of a combined SYTO 9 and propidium iodide solution (Thermo Fisher, Pittsburgh, USA) and incubated for 30 min at 30 °C. Then, the plates were rinsed again with water and mounted on microscopy slides. Biofilm samples were microscopically analyzed on an Axio Imager 2 fluorescence microscope, which is especially designed for material surface analysis (Carl Zeiss, Jena, Germany). The fluorophore SYTO 9 was visualized with an excitation of 470 nm while propidium iodide was visualized with an excitation of 558 nm. The emission settings for the used filters were 509 nm for SYTO 9 and 570 nm for propidium iodide. The images were then processed using the ZEN 3.3 blue software^101^. The green and red fluorescence intensities within the different field of views were then quantified and set into correlation using ImageJ software (Version: 2.0.0-rc-69/1.52n)^98^.

## Supplementary Information

Supplementary Information.
