# Supplementary material for: High-throughput synthesis of CeO2 nanoparticles for transparent nanocomposites repelling Pseudomonas aeruginosa biofilms
Source: Sci Rep. 2022 Mar 10;12:3935. doi: 10.1038/s41598-022-07833-w (PMC8913809; doi:10.1038/s41598-022-07833-w)
Supplement: Supplementary file 1 — Supplementary Information. [file 41598_2022_7833_MOESM1_ESM.docx]

**Supplementary Information**

High-Throughput Synthesis of CeO_2_ Nanoparticles for Transparent Nanocomposites Repelling *Pseudomonas aeruginosa* Biofilms

Massih Sarif,^1, †^ Olga Jegel,^1, †^ Athanasios Gazanis,^2^ Jens Hartmann,^1^ Sergi Plana-Ruiz,^1,3^ Jan Hilgert,^1^ Hajo Frerichs,^1^ Melanie Viel,^1^ Martin Panthöfer,^1^ Ute Kolb,^1,3^ Muhammad Nawaz Tahir,^4,5^ Jörg Schemberg,^6^ Michael Kappl,^7^ Ralf Heermann^2*^ and Wolfgang Tremel^1^*

^1^ Department Chemie, Johannes Gutenberg-Universität Mainz, Duesbergweg 10-14, D-55128 Mainz, Germany

^2^ Johannes-Gutenberg-Universität Mainz, Institut für Molekulare Physiologie, Mikrobiologie und Biotechnologie, Hanns-Dieter-Hüsch-Weg 17, D-55128 Mainz, Germany

^3^ Department of Materials and Geoscience, Technical University Darmstadt, Petersenstrasse 23, D-64287 Darmstadt, Germany

^4^ Chemistry Department, King Fahd University of Petroleum and Materials, Dhahran 31261, Kingdom of Saudi Arabia

^5^ Interdisciplinary Research Center for Hydrogen and Energy Storage (IRC-HES), King Fahd University of Petroleum and & Minerals, Dhahran 31261, Saudi Arabia

^6^ Institut für Bioprozess- und Analysenmesstechnik e.V., Rosenhof, D-37308 Heilbad Heiligenstadt, Germany

^7^ Max-Planck-Institute for Polymer Research, Ackermannweg 10, D-55128 Mainz, Germany

† these authors contributed equally to this work

**Table S1.** General information concerning the Rietveld refinement.

| General information | Description |
| --- | --- |
| Diffractometer  Detector | Stoe Stadi P  Dectris Mythen 1k |
| Wavelength (Å) | 0.7093 (MoKα_1_) |
|  |  |
| Temperature (K)  Program | 298  TOPAS Academic 6.0 |
|  |  |
| Number of parameters  Space group (cerianite) | 17  Fm-3m |
| Wyckoff positions | Ce: 4a, O: 9c |

**Table S2**. Information concerning the Rietveld refinement.

| Sample | R_wp_ | Cell parameter (Å) | Crystallite size (nm) |
| --- | --- | --- | --- |
| MF RT | 4.36 | 5.418(1) | 5.86(7) |
|  |  |  |  |
| MF 185 °C | 4.21 | 5.423(1) | 5.83(5) |
|  |  |  |  |
| MF 500 °C | 4.44 | 5.4141(2) | 16.2(1) |
|  |  |  |  |
| MF 800 °C | 8.70 | 5.41678(2) | 71.5(4) |
|  |  |  |  |
| Batch RT | 4.58 | 5.412(1) | 3.15(2) |
|  |  |  |  |
| Batch 185 °C | 4.28 | 5.417(2) | 3.78(3) |


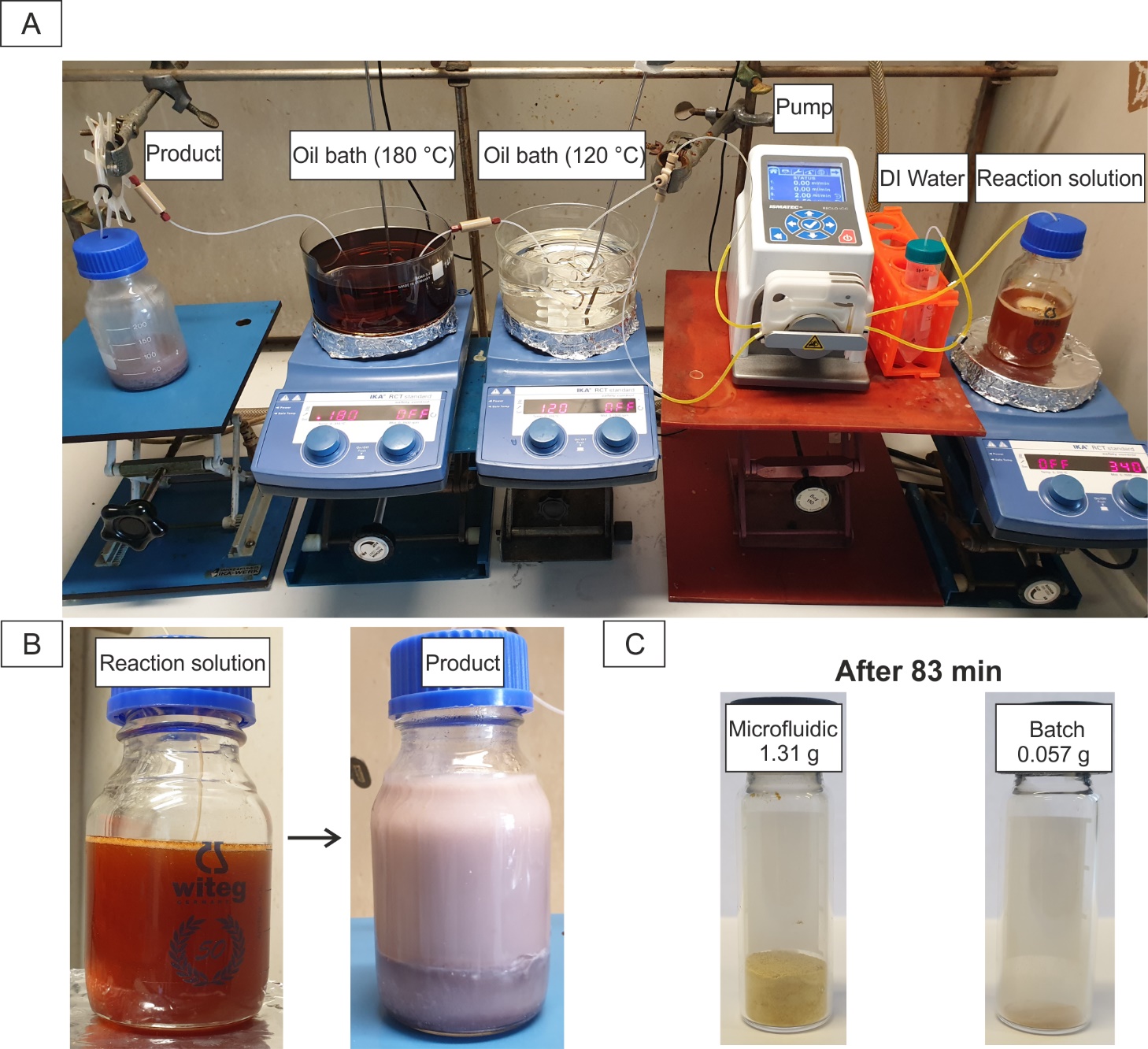


**Figure S1.** (A) Setup for the segmented flow, (B) comparison of the reaction solution and the product. (C) CeO_2_ NPs after 83 minutes in the segmented flow and in the batch process.


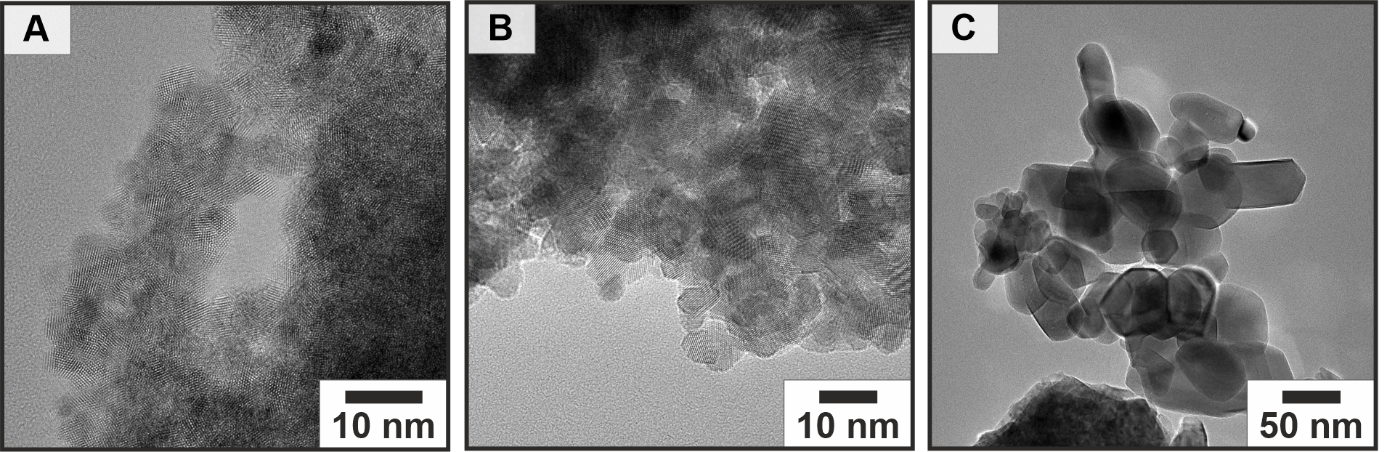


**Figure S2.** HRTEM images of CeO_2_ NPs obtained under segmented flow conditions and annealed for 5 h at (A) 185 °C, (B) 500 °C and (C) 800 °C.


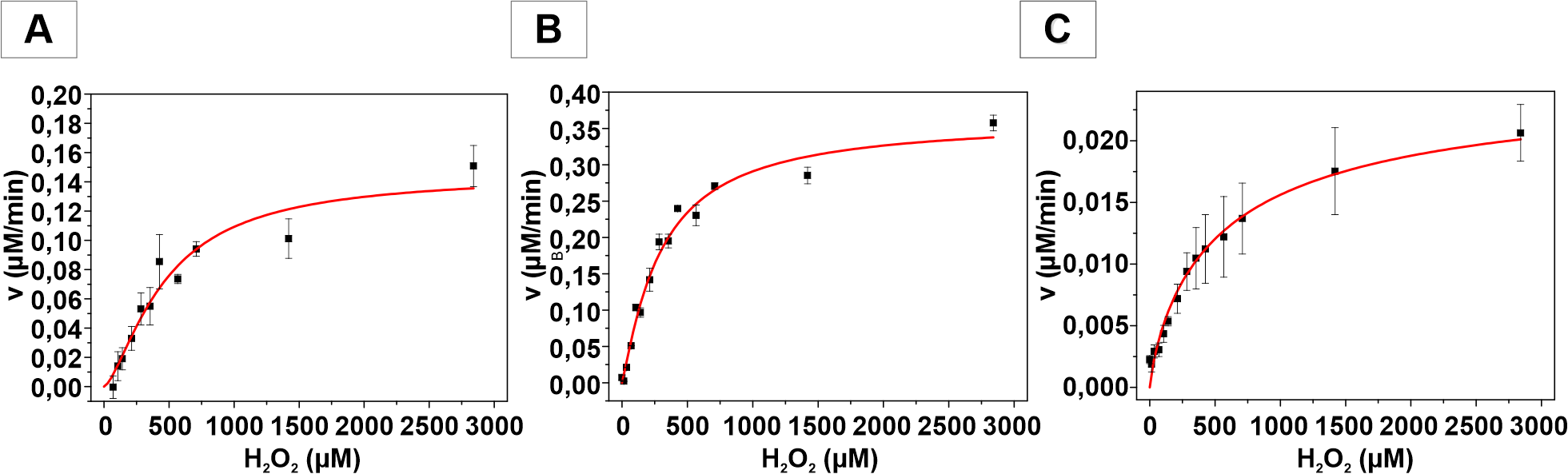


**Figure S3.** Michaelis-Menten kinetics for H_2_O_2_, with the corresponding Hill fit (red line) for the CeO_2_ NPs obtained from the segmented flow (A) annealed at 185 °C, (B) 500 °C and (C) 800 °C.


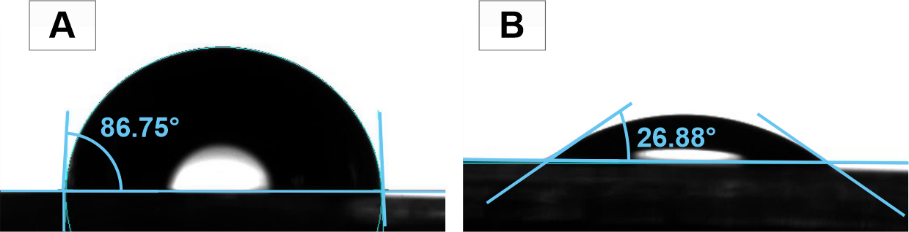


**Figure S4.** Contact angle measurements of polycarbonate with water (A) before and (B) after oxygen plasma treatment.


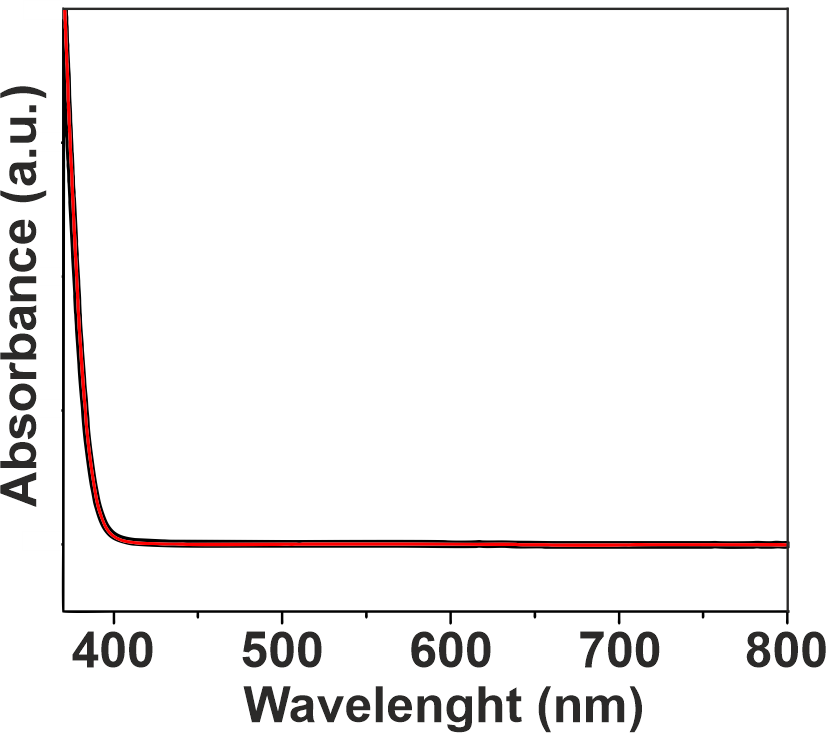


**Figure S5.** UV-VIS absorption spectra of polycarbonate before (black) and after coating (red) with CeO_2_ NPs.
